# Use of a long-acting substitute in detoxification from benzodiazepines: safety (accumulation) problems and proposed mitigation procedure

**DOI:** 10.1007/s00228-022-03388-x

**Published:** 2022-09-17

**Authors:** Anna Basińska-Szafrańska

**Affiliations:** Institute of Psychiatry and Neurology in Warsaw, Al. Sobieskiego 9, 02-957 Warsaw, Poland

**Keywords:** Benzodiazepine addiction, Detoxification, Elimination time, Diazepam accumulation, Anti-accumulation paradigm, Serum BZD tracking

## Abstract

**Objective:**

In the majority of approaches, detoxification of patients with benzodiazepine (BZD) addiction is preceded by conversion to long-acting BZDs. Resulting BZD accumulation, however, is neither monitored nor prevented. An unrecognized shift of the key low-concentration phase beyond the nominal treatment period may underlie delayed unassisted crises and treatment failures. This open, single-arm, semi-naturalistic study examines the anti-accumulation paradigm to minimize the high-concentration treatment phase and to regain time for medical assistance during the low-concentration phase.

**Methods:**

In 133 of 165 patients with BZD dependency, after conversion to diazepam by titration up to the satiation state, the loading dose and satiating concentration were recorded. The subsequent anti-accumulation procedure consisted of aggressive daily dose reductions under laboratory feedback (serum BZD concentration, radioimmunoassay) until accumulation stopped. The final overaccumulation ratio (OA) and maintenance-dose/loading-dose ratio (MTN) were estimated. The post-conversion peak-concentration/loading-dose ratio was illustratively compared with the concentration/dose ratio in 32 long-term diazepam users demonstrating the natural plateau.

**Results:**

Despite gender- and age-related differences in loading and maintenance doses and in satiating and peak concentrations (higher in younger and male patients), their quotients remained similar. The MTN ratio had an average value of 0.29 and a median value of 0.25, with OA ratios of 1.54 and 1.39, respectively. The concentration/dose ratio was approximately 3 times lower than that in regular diazepam users. With effective elimination starting (on average) from the 6th day, the treatment, including post-elimination recovery, lasted on average 52 days.

**Conclusions:**

The MTN values show how harmfully popular tapering schedules intensify and extend the high-concentration stage during alleged detoxification, leading to unrecognized delays in elimination, and delayed withdrawal crises. The common errors are discussed. An individual MTN, estimated from laboratory feedback (the anti-accumulation paradigm), expeditiously moves patients to the onset of actual detoxification. This action regains time to maintain medical assistance until treatment is properly completed.

**Supplementary Information:**

The online version contains supplementary material available at 10.1007/s00228-022-03388-x.

## Introduction

In some patients with benzodiazepine (BZD) addiction, withdrawal symptoms emerge late/long after drug discontinuation [1–4]. To date, these delayed/protracted crises have been attributed to individual inertia of biological readaptation processes. However, the first large-sample study using serum BZD concentration tracking revealed that the timing of the last of consecutive withdrawal crises, which was often (45%) the strongest one, was correlated with the time at which the concentration reached zero [5]. These results are in line with both older small-sample studies on BZDs [6–8] and modern views on discontinuing medications at all [9]. It has also been shown that a BZD concentration of zero occurs with a variable but usually marked delay after drug withdrawal [5]. The related crisis, seemingly late and surprising to the patient, may then trigger a relapse.

Thus, discharge from a detoxification ward, if based solely on the clinical criterion (of the patient’s satisfactory post-withdrawal condition) and ignoring elimination status, often turns out to be premature [10]. However, such discharge often completes many (4–8 on average) weeks of the tapering procedure [11, 12]. It may be challenging to convince a patient to stay under constant medical supervision until elimination is complete despite seemingly successful drug discontinuation. Therefore, if detoxification is to be concluded only after complete elimination but is not to take excessive time, the procedure should be optimized to reconcile the two requirements.

Some determinants of delayed elimination relate to how detoxification is performed [5]. One determinant is the moment when the concentration decrease begins. This may be delayed by the continuing accumulation of a long-acting substitute BZD, which is routinely introduced to replace previously used drugs to prevent rapid concentration drops. Another determinant is the level at which elimination starts (the maximal accumulation level). Optimization of the procedure preceded by a switch to a long-acting BZD should therefore be focused on these two initial conditions.

Routinely, these two conditions are not controlled. Physicians know that accumulation, even when left unattended, is limited. The starting dose of a substitute BZD significantly accumulates only for the first five elimination half-lives (T_1/2_). However, when considering diazepam which is typically used (T_1/2_ = 36–200 h [13]), this means up to 6 weeks of accumulation progress. During this time, concentration reaches the level (from which the patient needs then to be detoxified) proportional to D_*_T_1/2_/t, where D is the established substitute dose and t is the inter-dose interval. If D represents the loading (satiating) dose, its daily repetitions may result in a manifold increase over the satiating concentration level.

Despite the simplicity of the formulas, individual accumulation results remain unknown due to the wide range of diazepam T_1/2_ values. Thus, contrary to what is tacitly assumed (but not measured) while forcing scheduled dose regimens [14], the serum BZD plateau not only varies greatly between patients but also tends to be well above the optimal (satiating but not higher) level (Table 1).

**Table 1:** Illustrative evidence of overaccumulation (OA) features. The set of all cases identified by the author while reviewing the hospital archives, at the search oriented on inpatients detoxified in a physician-arranged manner but with additionally serum BZD checks at the satiation state and the second time, incidentally, during the tapering course. Within the small sample meeting the search criteria, the frequency of overaccumulation is high

ID	Sequence of daily doses [mg] to satiation	Satiating concentration C_SAT_ [ng/ml]	Sequence of daily doses [mg] following satiation	Resulting concentration C_ACC_ [ng/ml]	OA ratio (C_ACC_/C_SAT_)
1	50–60	543	60–55﻿–55–55﻿–50–50﻿–50–45﻿–45–45﻿–40–40﻿–40	2250	4.14
2	15–20	181	20–15﻿–15–15﻿–10	824	4.55
3	10	596	10–10﻿–10–10	959	1.61
4	50–60	820	60–55﻿–55–50﻿–45–40	1499	1.83
5	20–20	623	20–17﻿–17–17﻿–15–12﻿–12–10	1576	2.53
6	50	981	30–30﻿–30	1961	2.00
7	50	1499	50–30	2237	1.49
8	40	257	30–25	722	2.81
9	40	694	40–25﻿–20–15	1219	1.76

Actively stopping accumulation to start detoxification earlier and from a reasonable concentration level requires reducing the dose by some factor dependent on the individual T_1/2_. In the past, it was proposed to cut the loading diazepam dose to 50% [15] or even 40% [16] of the calculated equivalent. This uniform approach, however, resulted in one delirium case [16]. The customized maintenance dose, in turn, is hard to estimate without concentration measurements. However, after a few interesting but small-sample studies [6–8, 16], concentration tracking during detoxification was abandoned and has remained so for decades. Currently, serum BZD measurements are not taken for purposes other than toxicology or checking patient cooperation, or such measurements are deemed unjustified [17]. Moreover, some researchers have suggested prolonging each tapering stage until the assumed (not checked) new concentration steady state is reached [18]. While this approach is warranted in the low-concentration phase, it is detrimental at the beginning of detoxification. The widely allowed undisturbed (and unmeasured) accumulation of a substitute BZD is irrelevant and harmful because it (a) may reach excessive levels over the satiation concentration, (b) drives the adaptation process in the direction opposite to the treatment goal, (c) significantly delays the start of the effective elimination and raises the start level, and (d) shifts the critical low-concentration phase beyond the time period of scheduled medical assistance [10].

The present study addresses both safety and optimization issues, focusing on actively counteracting overaccumulation following successful substitution. An unknown individual maintenance dose will be empirically determined and controlled based on laboratory feedback.

The anti-accumulation paradigm was developed as an accessory action during the study on the BZD elimination course and related withdrawal crises [5]. It was intended to stop accumulation as hindering elimination assessment. In the present study, it became the main tool restoring physicians’ control over the entire detoxification process. By abbreviating the unsafe and irrelevant high-concentration stage (exceeding the satiating level), it is expected to shift the core low-concentration stage earlier and win time to trace it to its end. This open, single-arm, semi-naturalistic study aims to quantify the method and the duration of treatment with its use while requiring medical assistance beyond elimination completion. The maintenance-dose factor and its range, disclosed in the study, are intended to provide a perspective for the evaluation of widely used treatment regimens.

## Method

### Patients

A total of 165 patients (Supplement A) meeting the International Classification of Diseases (ICD)-10 criteria for benzodiazepine addiction [19] were included in the study during their treatment in the detoxification unit.

In this open, single-arm, semi-naturalistic study, the patients were recruited in a natural order of admissions with their informed consent. The study was not limited to patients with no coexisting somatic and psychiatric conditions. In contrast, as typical patients with BZD dependency, they often reported a history of insomnia, anxiety or mood disorders, or alcohol abuse. However, stable abstinence (no withdrawal symptoms) from substances other than BZDs and a stable clinical state were required. Therefore, the patients maintained their basic treatment, but drugs significantly influencing BZD metabolism were noted (Supplements B, C).

### Procedure

The detoxification procedure included 4 stages [5].

1. Substitution
  Following popular approaches, the detoxification was preceded by replacing the formerly used BZDs with a standard long-acting BZD. Diazepam was used due to its good traceability in the available laboratory tests, including the active metabolites. The conversion was a one-stage process. The initial (loading) oral dose of the substitute was completed using a titration procedure [20] with inter-dose intervals of 1–2 h up to the patient’s (reported and observed) satiation state. That clinically determined satiation state was then quantified [5] by individual self-scoring on the Clinical Institute Withdrawal Assessment Scale-Benzodiazepines (CIWA-B) questionnaire [21] and by corresponding serum BZD concentration level, thus yielding individual baselines for both clinical state and concentration. The baselines were introduced due to the limited informative value of raw individual serum BZD levels [17, 22] and raw CIWA-B self-report scores. For the serum BZD measurements, the assay typically available was used (SBENZ immunoassay/COBAS Integra 400 plus analyser, Roche Diagnostics, limit of detection (LOD) 3 ng/mL; precision 5.5% CV; accuracy: 100% for negative samples, 100% for gas chromatography/mass spectrometry (GC/MS)-positive samples; 8/74 samples tested positive at GC/MS-negative results) [23].
2. Anti-accumulation paradigm
  Given the satiating dose and the satiating (baseline) serum BZD concentration, actions were introduced to prevent further accumulation of the substitute. The daily doses were reduced daily, beginning with subtraction of 1/4 to 1/3 of the loading dose (bringing it to approximately 70%). The next tapering steps varied as estimated roughly from the laboratory feedback. The serum BZD measurements were performed daily in the morning (approximately 8 h after the previous dose) until accumulation stopped. Non-monotonic fluctuations < 10% or monotonic fluctuations < 5% between at least 3 successive samples were considered a (quasi-)plateau. The peak accumulation and the day of its occurrence, counted from the start of treatment, were noted, and the corresponding diazepam dose was estimated.
3. Elimination
  Further dose tapering started effective elimination. The tapering became slower and flexibly adjusted to the reported and observed patient’s clinical state (the symptom-driven approach [24–26]). However, constant medical assistance was maintained after drug withdrawal to the end of the elimination process. Any marked change in the patient’s condition was quantified (CIWA-B), and days of local extrema in the CIWA-B-score series marked exact timing of successive crises [5] (in this study used for the last crisis only). If needed, accessory medication was allowed (Supplement D). At this stage, serum checks (every 3–7 days) served to track the elimination course. A serum BZD decline below the trackable level (< 3 ng/ml) was tentatively adopted as the end of the stage.
4. Post-elimination readaptation
  That stage was intended to assist patients with their withdrawal symptoms up to the patients’ adaptation to the abstinence (approaching the CIWA-B baseline). Up to this point, the adjunct medication, adjusted to currently dominating ailments, was maintained. Blood samples were taken only at random times, as abstinence controls.

## The non-anti-accumulation paradigm (NAA) variant

For ethical reasons, there was no formal control group in this study. Substitution without counteracting accumulation, although commonly used until now, would have had to be performed under parallel concentration tracking (double-blind), thus requiring a deliberate tracing of undisturbed harm: overaccumulation (and possible toxemia symptoms) and delayed detoxification start. However, in some patients, diazepam was the drug of abuse before admission. These patients were not submitted to the anti-accumulation paradigm as those who had already reached, or were approaching, the natural concentration plateau. Before they underwent detoxification beginning with stage 3, their entrance data were recorded for illustrative comparison with the post-conversion data in the study group. Their declared doses of diazepam were verified by double concentration check: at the entrance and after 1–2 days of their use in the ward.

## Data elaboration

The detoxification course, although flexible, could be quantified by some essential variables. These are as follows:

d_SAT_, C_SAT_: the initial (loading) diazepam dose and the resulting satiating (baseline) BZD concentration
D_ACC_, C_ACC_: the day (counted from the beginning of the treatment) and the level of maximal serum BZD accumulation
OA: the overaccumulation ratio C_ACC_/C_SAT_
d_MTN_, MTN: the maintenance dose and ratio (MTN = d_MTN_/d_SAT_)
D_W_: the withdrawal day (next day after the last administered dose)
D_E_: the elimination day (the day of trackable elimination completed)

The results were compared between male and female patients and between those who received elimination-modifying medications and those who did not. The relationships of the treatment-related elimination data with the clinical data (last withdrawal crisis timing D_LAST_ and total treatment duration) were examined.

Due to the asymmetrical distribution of the data, apart from the average and the standard deviation (SD), which are presented for information only, the median value and the interquartile range are presented. For analyses, non-parametric tests were applied: the Mann–Whitney U test for intergroup comparisons and Spearman’s rank test for correlations between relevant patient- and detoxification-related data. The tests were performed using Statistica version 13.3 [27].

## Results

### A. The NAA group

Among the patients included, 32 had been regular diazepam users before admission. Twelve of them used the BZD metabolism modifiers: 6 -  carbamazepine, and 6 -  valproates. In each case, the entrance and confirmatory concentration results were similar (difference < 5%), confirming the patient’s declaration. Their entrance data (using the second concentration check) were collected in Table 2 and compared with the study group post-conversion data (Table 4). Their stage 3 data were not analysed. Eight of them left the study right after diazepam withdrawal, arguing that they felt well and considered their detoxification completed.

**Table 2:** Patients who were excluded from the anti-accumulation paradigm (the NAA subgroup) as taking diazepam regularly prior to admission and already at a natural concentration plateau. For a comparison with the study group (SG), see Table 4

**No**	**AGE**	**Dose recently maintained [mg]**	**Serum BZD at admission [ng/mL]**
1	35	20	1200
2	30	85	1433
3	49	10	1355
4	50	5	413
5	68	5	509
6	52	5	1060
7	47	40	1619
8	28	40	1440
9	63	30	2000
10	35	5	830
11	59	10	1029
12	27	10	733
13	51	50	4441
14	47	15	1447
15	72	5	435
16	43	20	1340
17	38	30	1141
18	29	45	2032
19	50	10	233
20	81	10	739
21	41	15	1760
22	50	47.5	4760
23	63	5	168
24	39	45	1099
25	56	100	1912
26	49	20	1531
27	62	10	989
28	78	5	918
29	43	12	1632
30	42	20	894
31	72	2.5	179
32	32	50	1915
**Average**	**49.4**	**24.4**	**1350**
**Std Dev**	**14.8**	**23.7**	**1006**
**Median**	**49**	**15**	**1171**
**1. Quartile**	**38.5**	**8.5**	**807**
**3. Quartile**	**60.5**	**40**	**1625**

Bolding was introduced to graphically differentiate between individual and collective (group) data

### B. The study group

#### Stage 1:

In the remaining 133 subjects, the substitution procedure was well tolerated and usually did not exceed the first 24 h. The satiation state was achieved at the average loading diazepam dose d_SAT_ = 37.4 (SD 29.0) or median 30 (the interquartile range 15–55) mg, at corresponding serum BZD concentration C_SAT_ = 526 (418) or median 397 (260–685) ng/ml. Both d_SAT_ and C_SAT_ were higher in men and younger patients (Table 3).

**Table 3:** The substitution- and accumulation-stage data. Although doses and concentrations vary with patient sex and age, the derivative factors OA and MTN remain similar

Variable	Average (SD)	Median (interquartile range)	Females Males	Inter-sex difference (Z, p)	Correlation with age (rho, p)
d_SAT_ [mg]	37.4 (29.0)	30.0 (15.0–55.0)	27.5 (15.0–50.0) 40.0 (20.0–60.0)	− 2.26, 0.023	− 0.45, < 0.0000005
C_SAT_ [ng/ml]	526 (418)	397 (260–685)	350 (190–631) 547 (305–826)	− 3.15, 0.002	− 0.19, 0.025
C_ACC_ [ng/ml]	769 (587)	561 (368–1043)	460 (247–788) 792 (453–1298)	− 2.96, 0.003	− 0.26, 0.003
OA	1.54 (0.55)	1.39 (1.20–1.68)	1.30 (1.12–1.51) 1.39 (1.23–1.71)	ns	ns
d_MTN_ [mg]	9.3 (9.3)	6 (3–10)	5.0 (2.0–10.0) 8.0 (5.0–15.0)	− 2.44, 0.015	− 0.35, 0.00004
MTN	0.29 (0.21)	0.25 (0.13–0.40)	0.25 (0.14–0.38) 0.25 (0.13–0.41)	ns	ns

#### Stage 2:

Recurrent daily dose reduction based on laboratory feedback resulted in cessation of further BZD accumulation on the 5th–6th day of treatment (D_ACC_ = 5.6 (3.0) on average, or median 6 (4–7)). Up to this day, the concentration increased to an average C_ACC_ of 769 (587) or a median of 561 (368–1043) ng/ml, with higher values in men and younger patients; the average and median C_ACC_ corresponded to OA ratios of 1.54 (0.55) and 1.39 (1.20–1.68), respectively, with no sex- or age-related differences (Table 3). The accumulation stopped when the daily dose was reduced to an average of 9.3 (9.3) mg or a median of 6 (3–10) mg, with higher values in men and younger patients, or at an average MTN factor of 0.29 (0.21) or median MTN factor of 0.25 (0.13–0.40) regardless of sex and age.

There was a considerable bulk of patients receiving carbamazepine (58%) or valproates (24%), facilitating or slowing BZD elimination, respectively. Their accumulation-stage data did not significantly differ from those in the other patients.

The illustrative comparison between the relevant stage 1 and stage 2 data in SG group and the NAA group entrance data (Table 4) revealed a much higher C_ACC_/dose ratio in the latter group.

**Table 4:** Accumulation counteraction results in the study group (SG) compared with the natural steady-state data from the primary diazepam users’ group (non-anti-accumulation, NAA). The ratio of steady (peak) concentration C_ACC_ to the initial daily diazepam dose used (loading dose d_SAT_ in the SG or declared dose in the NAA group) is approximately 3 times higher in the latter group, at the 2-fold lower median daily dose

Variable	Average (SD)		t, p	Median (Interquartile range)		Z, p
	SG 40.4% of men	NAA 54.5% of men		SG 40.4% of men	NAA 54.5% of men	
Age (years)	51.9 (15.6)	49.4 (14.8)	ns	52 (41–63)	49.0 (38.5–60.5)	ns
Initial dose [mg]	37.4 (29.0)	24.4 (23.7)	2.31, 0.022	30.0 (15–55)	15 (8.5–40)	2.80, 0.005
C_SAT_ [ng/ml]	526 (418)	unknown	-	397 (260–685)	Unknown	-
C_ACC_ [ng/ml]	769 (587)	1350 (1006)	− 4.23, 0.00004	561 (368–1043)	1171 (807–1625)	− 3.84, 0.0001
OA = C_ACC_/C_SAT_	1.54 (0.55)	unknown	-	1.39 (1.20–1.68)	Unknown	-
C_ACC_/initial dose [10^−3^/ml]	28.5 (29.8)	79.9 (48.1)	− 7.22, < 0.0000005	20.5 (12.3–32.1)	73.6 (39.4–101.0)	− 6.46, < 0.0000005

Stages 3–4 were beyond the main scope of this study so their analysis is provided in Supplement E. In general, stage 3 completion was facilitated in the ‘carbamazepine’ group. The key D_E_ x D_LAST_ correlation (overall ρ = 0.62, *p* < 0.0000005), in the ‘carbamazepine’ group was comparable to that in the ‘non-modifier’ group, while it was the lowest in the ‘valproate’ group. These differences did not significantly influence stage 4 completion, as the readaptation process ended similarly in all groups.

All 133 patients completed the treatment. The entire procedure took an average of 52 (19) or a median of 51 (38–60) days. The length of stay correlated with elimination completion (ρ = 0.55, *p* < 0.0000005) and (as oriented to) the last crisis occurrence (ρ = 0.74, *p* < 0.0000005).

## Discussion

The postulate of concluding detoxification only after complete elimination remains crucial. This study confirmed the previously reported correlation in time between elimination completion and the last withdrawal crisis. The presented anti-accumulation paradigm reconciles this postulate with the demand of acceptable detoxification duration. The entire modified procedure in typical study group patients fits within the 5.5- to 8.5-week period, which is comparable to the traditional standards [11, 28].

Completion of the procedure within that time results from the contraction of the irrelevant high-concentration phase following substitution with a long-acting BZD. In this study, there was no control group directly demonstrating the actual time course of concentration changes when tapering was carried out in a manner deemed adequate by a physician blinded to laboratory results. However, if such a trial were available, the comparison would be based on the two parameters opening the effective elimination stage: its start level and its delay. These data are available herein, indirectly.

Considering the start level first, the mean maintenance dose revealed using the anti-accumulation paradigm turned out to be lower than 1/3 or even (median) 1/4 of the loading dose, and in 75% of patients it did not exceed 40%. The NAA group demonstrated a natural steady state, resulting from a constant dose, with a concentration/dose ratio approximately 3 times higher than concentration/loading dose ratio in the study group. By analogy, in the (absent) control arm, where a loading dose would be repeated for some days (a regular custom supported by popular tapering schedules), a similar concentration excess would be legitimately expected. The MTN value indicates that the commonly used detoxification schedules provide excessive amounts of diazepam, and the finding affirms that the data in Table 1 are not incidental. These unbiased (all available) detoxification records, with concentration measurement at satiation and with the second check incidentally imposed on a physician-arranged tapering procedure, contain no cases revealing a concentration decrease and only 2 cases with OA fitting within the interquartile range for the study group. In some other cases, the OA even exceeds 4.

Considering the delay in starting an effective elimination, the median maintenance dose of 1/4 of the initial daily diazepam dose, reached approximately the 6th day here, places patients already on an advanced tapering stage. In many tapering schedules, the same stage (from which detoxification actually begins) is delayed by weeks to months [18]. In the cases shown in Table 1, the second check might have fallen on the rising or descending concentration slope, but in all cases, the concentration was still over the satiation level.

Translated into clinical consequences, the excessive serum BZD levels (which constitute the first factor) are alarming in terms of safety. The NAA-group concentrations are congruent with those observed in diazepam users arrested for impaired driving [29]. Moreover, they may drive the adaptation process in the direction opposite to the treatment goal, which may contribute to readaptation problems later. The second factor, the delay of effective elimination, is even more clinically important. Weeks of delay while approaching the (median) ¼ of the initial diazepam dose waste most of the time (depending on a schedule) devoted to the entire detoxification procedure.

Elimination delay and elimination from overaccumulated concentrations cause a concentration inadequacy (inflation) in relation to the tapered doses. This concentration inertia, if unnoticed, mimics good withdrawal tolerance. Finally, it may shift the low-concentration phase beyond the nominal end of treatment [10], with crises culminating afterwards. The eight NAA patients who left the study after diazepam discontinuation did so because they felt well at all times or argued that they had already passed through their crisis (plausibly not the last one). Subsequent follow-up data on these patients are not available. The actual delay of the start and end of elimination during an “as-usual” tapering procedure, observed under a laboratory double-blind tracking regimen, would be extremely interesting data, but collecting such data without corrective interventions would be ethically controversial. Future research will approach this concept.

The mean relative maintenance dose estimated in this study should not be a basis for any new regimen detached from laboratory control. Its dispersion, as the inevitable consequence of the diazepam half-life spread, requires individual (laboratory) assessment. With the maintenance dose arbitrarily set to 40%, the vast majority of Harrison et al. patients benefited from the treatment, while some patients suffered withdrawal-related complications [16].

The long-term neglect of laboratory tracking of BZD detoxification, regardless of costs and poorer availability of tests in the past, might have resulted from observations that serum BZD concentration values do not translate into the amount of the substance ingested or into intoxication severity [22]. Indeed, patients differ not only in their BZD metabolism rates but also in their compartment capacities and the degree of drug tolerance they have developed. However, relative concentrations (referred to individual baselines) are reliable data.

The reluctance to use laboratory control resulted in strategies to otherwise increase the safety of the BZD detoxification process. Some manuals, instead of one-step substitution, propose running it in instalments [18], allegedly securing patients’ conversion with the unchanged part of the previous drug. Several steps take 1–2 weeks each, with the intention of reaching a steady PK and clinical state related to the current conversion stage. However, the alleged plateau, if it occurs by then, may have nothing to do with that individually needed. Since the substitute instalments are calculated from single-dose equivalency tables, they accumulate by unknown factor when repeated. Thus, OA may initiate at any conversion step. To lower that risk, some physicians administer an underestimated dose, assuming that a proper concentration will be reached with progressive accumulation. In this case, not only is the steady state unrelated to the satiating concentration, but it is also preceded by days of patient discomfort. Saturation by titration, which was shown to be effective in acute alcohol withdrawal treatment in the 1980s [20], condenses a similar procedure down to 1 day.

The 1-day titration also avoids errors following a direct recalculation (from tables) of the daily sum of short-acting doses into their substitute equivalents. Not only the PD equivalents may differ individually, but they are definitely not PK equivalents. Ignoring half-life differences results in OA at the beginning.

Furthermore, even a correctly titrated first-day (loading) dose of a substitute, if temporarily maintained for patient’s alleged “stabilization” [30], or if reduced too slowly, builds the OA by the individual, unknown factor. These two customs are other actions serving to increase physicians’ sense of security, with patient applause. Longer-lasting ‘stabilization’, due to multiple repetitions of the established loading dose, will drive to a concentration excess, as discussed before. The initial slow/small tapering steps, which are intended to adapt the patient to declining drug content in the body [18], in view of the revealed MTN ratio adapt to overaccumulation instead.

Crucially, it is not clear why patients should adapt to a given dose and not to a given concentration. The anti-accumulation paradigm, promoted herein, not only rationally sets the initial conditions for elimination but also, if needed, provides a concentration plateau for patient adaptation.

Thus, safety manoeuvres replacing laboratory control do not protect against errors but actually contribute to them by extending the high-concentration procedures in intensity and time.

Concerning actual patient safety, the median 5 days to stop overaccumulation in this study resulted from a conservative sequence of smaller daily steps. That conservatism resulted from the awareness that (a) in the first 1–2 days, elimination of the formerly taken (shorter-acting) BZDs occurs, (b) shifts between the compartments are possible, and (c) some patients are fast metabolizers.

Certainly, the method has limitations. Immunoassays (IAs) are tests with a lower quantitative precision than GC/MS or high-performance liquid chromatography. Moreover, they often measure serum BZD in bulk (all BZDs together), while consecutive samples contain the evolving mix of diazepam and its BZD metabolites, resulting in molecular mass changes. These issues have been discussed in detail [5, 10], with the conclusion that for the purposes of the presented method, IAs provide sufficient accuracy and an optimal quality/cost ratio. This is decisive for the applicability of the anti-accumulation paradigm in the everyday detoxification ward routine.

The C_SAT_ for diazepam is approximate also because residues of primary BZDs, proportion unknown, may have contributed to the satiation state but could not be measured with the available method. Also, interdose intervals during the titration procedure were shorter than in the next days (daily doses typically divided into 2–3 parts). These inaccuracies, however, make the ‘small MTN’ result even underestimated.

Furthermore, the necessary large dose-reducing steps result in lower precision of empirical d_MTN_ estimation. However, in practice, the proper goal of anti-accumulation paradigm is not to precisely measure d_MTN_ itself but to achieve accumulation cessation. Laboratory feedback provides rough but functional adjustment of the diazepam maintenance dose as it automatically corrects for not only the patient’s individual metabolism rate but also for contributions (known or unknown) of any metabolism modifiers.

Neither carbamazepine nor valproates affected the stage 2 duration. Theoretically, they could modify the d_MTN_ and MTN itself, but in this sample, the differences were non-significant. For carbamazepine, this may be due to self-limiting activity resulting from induction of its own metabolism [29, 31] and the only facilitation effect could be observed in the elimination stage (Supplement E). For the ‘valproate’ group, lack of effect on d_MTN_ coincides with a failed replication of the elimination-stage study results (larger sample) [5], where elimination tended to be the longest and the key D_E_ x D_LAST_ correlation was the strongest in this group. This requires further studies considering plausible co-factors.

## Conclusions

Small or/and slow tapering steps make sense only when the concentration is confirmed to be declining. Moreover, they are definitely justified in the low-concentration stage, when each dose reduction poses a challenge for the adaptation process, and the actual, dose-adequate steady state allows for gradual adaptation. The maximum reduction of the irrelevant high-concentration phase using the anti-accumulation paradigm is precisely to regain time to assist this process.

## Supplementary Information

Below is the link to the electronic supplementary material.

Supplementary file1 (DOCX 6 KB)

Supplementary file2 (DOCX 6 KB)

Supplementary file3 (DOCX 6 KB)

## Data Availability

The datasets generated or analysed during the current study are not available publicly due to local data protection regulations but available from the author on reasonable request.
