# Supplementary material for: Use of a long-acting substitute in detoxification from benzodiazepines: safety (accumulation) problems and proposed mitigation procedure
Source: Eur J Clin Pharmacol. 2022 Sep 17;78(11):1833–41. doi: 10.1007/s00228-022-03388-x (PMC9546953; doi:10.1007/s00228-022-03388-x)
Supplement: Supplementary file 1 — Supplementary file1 (DOCX 6 KB) [file 228_2022_3388_MOESM1_ESM.docx]

Supplement A

Characteristics of the group entering the study. In some patients more than one co-morbid disorders could have been diagnosed.

Abbreviations: SD – standard deviation, ns – non significant difference. d. - disorder

|  | Women | Men | Total | Differences |
| --- | --- | --- | --- | --- |
| Participants (%)  Drop-outs (%) | 71 (43)  2 (2.8) | 94 (57)  6 (6.4) | 165 (100)  8 (4.8) |  |
| Age:  average (SD)  median (interquartile)  Patients > 65 years | 53.1 (15.3)  51 (44-62)  12 | 50.3 (15.2)  48 (41-59)  9 | 51.5 (15.5)  50 (41-61)  21 | ns  ns |
| Years of BZD addiction: average (SD)  median (interquartile) | 11.5 (7.8)  10 (7-18) | 11.1 (9.7)  9 (4-15) | 11.2 (8.6)  10 (4-15) | ns  ns |
| Co-morbid disorders (cases):  alcohol dependence  anxiety disorders:  - panic anxiety d.  - generalized anxiety d.  - other/mixed anxiety d.  mood disorders:  - bipolar disorder  - major depressive d.  - other depressive disorders  personality/behaviour d.  primary insomnia | 18  5  22  5  2  16  6  9  5 | 35  13  13  12  13  10  18  12  9 | 53  18  35  17  15  27  24  21  14 |  |
