# Supplementary material for: Use of a long-acting substitute in detoxification from benzodiazepines: safety (accumulation) problems and proposed mitigation procedure
Source: Eur J Clin Pharmacol. 2022 Sep 17;78(11):1833–41. doi: 10.1007/s00228-022-03388-x (PMC9546953; doi:10.1007/s00228-022-03388-x)
Supplement: Supplementary file 2 — Supplementary file2 (DOCX 6 KB) [file 228_2022_3388_MOESM2_ESM.docx]

Supplement B

The most frequent psychiatric medications accepted and continued throughout the study. In particular, the anticonvulsive agents, formerly introduced, could not be withdrawn at the detoxification onset.

| Medication | Cases | Condition | Application |
| --- | --- | --- | --- |
| valproates | 38 | bipolar mood disorder  other mood disorders  benzodiazepine dependence | mood stabilizer (normothymic)  adjunct medication  anticonvulsant (securing against witdrawal seizures) |
| carbamazepine | 82 | mood disorders  personality/behavioral d.  benzodiazepine dependence | mood stabilizer, adjunct medication  adjunct medication  anticonvulsant, adjunct medication |
| lamotrigine | 3 | mood disorders | mood stabilizer |
| trazodone | 12 | mood disorders | antidepressant |
| venlafaxine | 16 | anxiety disorders  mood disorders | anxiety treatment  antidepressant |
| sertraline | 5 | anxiety disorders  mood disorders | anxiety treatment  antidepressant |
| es- or citalopram | 11 | anxiety disorders  mood disorders | anxiety treatment  antidepressant |
| tianeptine | 5 | mood disorders | antidepressant |
| mianserin | 15 | mood disorders | antidepressant |
| agomelatine | 10 | mood disorders | antidepressant |
| olanzapine | 7 | bipolar mood disorder  depressive mood disorder | anti-manic agent  adjunct and sleep-promoting drug |
| quetiapine | 34 | bipolar mood disorder  depressive mood disorder | anti-manic agent  adjunct medication |
| aripiprazol | 6 | bipolar mood disorder | anti-manic agent |

Supplement C.

The most frequent permanent medication of somatic illnesses in the study participants. as accepted and continued throughout the study.

| Medication | Condition |
| --- | --- |
| enalapril, ramipril  telmisartan  amlodipine  bisoprolol | arterial hypertension |
| rova- or atorvastatine | hypercholesterolemia |
| levothyroxine | hypothyroidism |
| doxazosine | prostatic hypertrophy  arterial hypertension |

Supplement D

The most frequent adjunct medication temporarily applied to alleviate BZD-withdrawal symptoms. The drugs were used on demand or temporarily applied on a regular basis.

| Medication | Symptoms |
| --- | --- |
| captopril  atenolol  propranolol  magnesium  ibuprofen  carbamazepine | elevated arterial pressure  tachycardia, elevated arterial pressure  tachycardia, tremor  tachycardia, tremor, increased muscle tension  headache, muscle pain  neuralgia, paresthesia |
| omeprazol  loperamide | gastrointestinal problems |
| hydroxyzine  promethazine  tiaprid  quetiapine | agitation, anxiety, sleep disturbances |
| trazodon  mianserin  mirtazapine  promazin | sleep disturbances |
