# Supplementary material for: Use of a long-acting substitute in detoxification from benzodiazepines: safety (accumulation) problems and proposed mitigation procedure
Source: Eur J Clin Pharmacol. 2022 Sep 17;78(11):1833–41. doi: 10.1007/s00228-022-03388-x (PMC9546953; doi:10.1007/s00228-022-03388-x)
Supplement: Supplementary file 3 — Supplementary file3 (DOCX 6 KB) [file 228_2022_3388_MOESM3_ESM.docx]

Appendix E

Elimination- and readaptation-stage data influenced by metabolism modifiers. Data from patients treated with carbamazepine (CBZ) or valproates (VAL) compared with the patients not receiving metabolism modifiers (NONE subgroup).

| Elimination data | | CBZ  tests vs. NONE | NONE | VAL  tests vs. NONE |
| --- | --- | --- | --- | --- |
| Elimination day (D_E_) | AVG (STD)  t, p  MED (quartiles 1-3)  Z, p | 34.1 (12.3)  2.38, 0.019  32.0 (26.0-39.0)  ns | 42.2 (17.9)  -  41 (28.0-52.0)  - | 38.6 (15.1)  ns  36.5 (27.5-51.0)  ns |
| Elimination duration  (D_E_—D_ACC_) | AVG (STD)  t, p  MED (quartiles 1-3)  Z, p | 28.5 (11.6)  2.32, 0.023  27 (20.0-34.0)  ns | 35.9 (16.6)  -  35 (21.5-46.0)  - | 33.8 (14.0)  ns  30 (24.0-45.0)  ns |
| Elimination after the drug withdrawal  (D_E_—D_W_) | AVG (STD)  t, p  MED (quartiles 1-3)  Z, p | 22.6 (12.8)  2.72, 0.008  20 (14.0-28.0)  2.73, 0.006 | 31.8 (15.5)  -  30 (19.5-37.0)  - | 27.3 (12.6)  ns  24 (17.5-36.0)  ns |
| Last crisis day  (D_LAST_) | AVG (STD)  t, p  MED (quartiles 1-3)  Z, p | 36.1 (17.9)  ns  34 (22.0-47.0)  ns | 43.7 (21.4)  -  39 (28.0-57.5)  - | 34.1 (17.3)  ns  29 (21.5-45.0)  ns |
| Treatment duration | AVG (STD)  t, p  MED (quartiles 1-3)  Z, p | 51.6 (18.4)  ns  51(39.0-60.0)  ns | 55.5 (21.8)  -  49.5 (41.0-73.0)  - | 52.7 (19.3)  ns  53 (35.0-59.5)  ns |
| Correlation  D_E_ x D_LAST_ | ρ (rho)  p | 0.67  <0.0000005 | 0.74  0.0002 | 0.45  0.01 |
| Correlation  D_E_ x treatment duration | ρ (rho)  p | 0.46  0.00003 | 0.65  0.003 | 0.70  0.000008 |
| Correlation  D_LAST_ x treatment duration | ρ (rho)  p | 0.73  <0.0000005 | 0.89  0.000001 | 0.67  0.00003 |
